# Reversing aging-like 3D genome disorganization in a *Drosophila* interphase model

**DOI:** 10.64898/2026.07.16.739007

**Published:** 2026-07-21

**Authors:** Alexey V. Onufriev, Junkai Zhang, Igor V. Sharakhov, Igor S. Tolokh

**Affiliations:** 1 Departments of Computer Science, Virginia Tech; 2 Department of Physics, Virginia Tech; 3 Center for Soft Matter and Biological Physics, Virginia Tech; 4 Department of Entomology, Virginia Tech; 5 University of Illinois, Chicago

## Abstract

Recent experimental evidence suggests that aging may arise from the progressive deterioration of the epigenetic landscape, while reversing the trend can result in cell and tissue rejuvenation. A mechanistic understanding of how restoration of a key component of this landscape – the 3D structure of the genome – can be accomplished is lacking. Here we investigate lamina-dependent disruption and recovery of the 3D architecture of the Drosophila melanogaster genome at TAD resolution (~ 100 kb), using a model of the entire nucleus; weakening of chromatin–lamina interactions mimics an aging-associated loss of chromatin organization. We characterize this loss using the Shannon entropy of appropriately normalized Hi-C contact matrices.

Our main finding is that lamina-depletion-induced increases in Hi-C map disorder, deterioration of chromosome territories, and cell-to-cell conformational heterogeneity are largely reversible when WT-like LAD–nuclear-envelope interactions are restored. The original and recovered conformational states of chromatin are nearly indistinguishable by bulk Hi-C contact matrix; the corresponding Pearson correlation coefficient is 0.999902.

The direct experimentally testable prediction is that restoration of functional LAD–lamina interactions will promote recovery of young/WT-like 3D chromatin architecture after lamina-dependent architectural disruption.

## 1 Introduction

Aging is a remarkably complex, multifactorial phenomenon that remains one of the grand unsolved problems of biology and medicine. Despite decades of research, there is no universally accepted theory of why organisms age or how this process is regulated [63, 12, 32]. Numerous mechanisms have been proposed, ranging from the accumulation of molecular damage to dysregulation of signaling pathways and loss of tissue homeostasis [47, 35]. Theories of aging can be divided into two broad, non-mutually exclusive groups: error-based and program-based theories. Error-based theories suggest that aging results from the accumulation of molecular and cellular errors and damage that repair mechanisms cannot fully counteract. In contrast, program-based theories suggest that aging is regulated by developmental genetic programs that, over time, become detrimental [21, 30], implying that the aging process emerges from genetic programs shaped by natural selection. Program-based theories of aging include the neuroendocrine theory [4] and the software design flaw hypothesis [20]. Among the most widely discussed explanations is the view that aging results primarily from the gradual accumulation of DNA damage and mutations, which over time compromise genomic integrity and cellular functions [60, 37]. Defects in DNA repair are also known to accelerate aging, and various forms of genomic instability are hallmarks of aged cells [94]. However, accumulating evidence, including healthy aging of cloned sheep [100] and normal lifespan of mice cloned up to 20 times [107], suggests that DNA damage alone may not fully explain the reversible and coordinated aspects of aging observed at the cellular and organismal levels. Recent work points to a complementary or even alternative perspective that aging, at least on certain timescales, may arise from the progressive loss of epigenetic information rather than from the mere accumulation of genetic lesions [99]. In this “information theory of aging”, the primary driver of functional decline is viewed as the gradual erosion of the chromatin-based regulatory architecture that maintains cellular identity. Conceptually, the information theory of aging is close to error-based theories that focus on information degradation, while incorporating features of program-based theories, such as interaction with developmental regulatory pathways. Epigenetic characteristics — such as histone modifications, DNA methylation patterns, and higher-order chromatin organization in 3D — encode regulatory information beyond the genome sequence itself. Their dysregulation can lead to aberrant gene expression programs reminiscent of aged or senescence-like phenotypes [25, 33, 96] even if the underlying DNA sequence remains largely intact.

During aging, cellular identity often becomes “fuzzy”, with aged genomes being in some ways less stable and more disordered than young ones [43, 61]. The tendency of chromatin to move toward more disordered, higher-entropy states with the progression of aging is perhaps not surprising. Much more surprising is the striking partial rejuvenation of aged cells through transient expression of Yamanaka factors [76, 64], which supports the notion that at least some features of aging are epigenetically encoded in chromatin marks and potentially reversible. These results are consistent with the possibility that changes in chromatin organization can contribute causally to aging- or senescence-linked transcriptional programs, rather than merely reflecting them. The mechanistic details of how this kind of reversal is achieved remain unclear. The expression of reprogramming factors in somatic cells initiates targeted chromatin remodeling prior to transcriptional changes via genome-wide changes in the promoter H3K4 methylation and gains of active H3K4me2 enhancer signatures [48]. Ectopic expression of Yamanaka factors Oct4, Sox2, Klf4 *in vivo* restores youthful gene-expression patterns and epigenetic features in aged tissues, partially reversing functional decline [110]. The observed blurring of the originally sharp histone modification patterns demonstrates that the aging process corrupts epigenetic information, thereby increasing its Shannon entropy [110]. While in principle there is no contradiction with the second law of thermodynamics, as the cell is an open system, orchestrating a precisely fine-tuned reduction of disorder in a complex system is a non-trivial task (going the other way is, usually, much easier).

One key layer of epigenetic information that has not been widely explored in relation to aging and, in particular, the potential for age reversal, is the 3D organization of the chromatin. How can a disorganized, presumably high-entropy state of aged chromatin be restored to its functional, lower-entropy configuration? In physics, the restoration of a lower entropy state can be effected easily by an appropriately chosen external factor: *e.g.*, freezing water into ice decreases the system’s entropy. Likewise, a magnetic field can act as an ordering agent, reducing randomness in the spin system and, thus, lowering its entropy as it drives the system toward a more ordered magnetic state. Not surprisingly, the situation in biology is expected to be more complex. The folding–unfolding transition in proteins is a good example to illustrate the point [27]. Any protein can be easily brought to a high entropy, disorganized (denatured) state by placing it into a denaturing environment [27], such as high temperature, urea or acidic pH [78], yet only for small globular proteins of relatively simple topology [85] this transition is truly reversible, that is the protein can be brought back to its original low entropy functional (native) state by simply removing the denaturing environment. This simple manipulation often fails to restore the original 3D shape of larger or more complex proteins, which generally require additional mechanisms such as chaperones to fold properly. The functional 3D configuration/topology of chromatin is no doubt exceedingly more complex than that of any protein, so finding a way to reverse its age-related chromatin disorganization is expected to be highly non-trivial.

The range of questions that must be addressed before organismal rejuvenation is understood is immense, reflecting the complexity of aging itself. Assuming that a reductionist approach may help here, as it did in the past with many other complex scientific problems, it is worthwhile to investigate simpler systems that have at least some of the key features of aging. The 3D chromatin organization is shaped by at least two major interaction types: (1) chromosome-chromosome interactions and (2) interactions between chromosomes and nuclear lamina, a dense meshwork of Lamin proteins underlying the inner nuclear membrane. Chromosome-chromosome (or chromatin) interactions are studied using Hi-C, a technique based on Chromosome Conformation Capture (3C) technology [57]. As a step toward a minimal model of aging-associated 3D genome disorganization, we focus here on interphase chromatin and on lamina-dependent architectural changes. Recent Hi-C experiments [101] demonstrated that such “interphase aging” of post-mitotic cells is meaningful: the experiments have revealed, in great detail, how chromatin organization in neurons evolves with age, clearly demonstrating significant changes in the chromatin structure in aging nuclei. When the brains of newborns, one-year-olds, ten-year-olds, thirty-year-olds, and eighty-year-olds human individuals were compared, ultra-long-range chromatin contacts and specific inter-chromosomal contacts were identified as markers of cerebellar granule cell age. It has been hypothesized that the restructuring of the 3D genome that occurs over the course of a human lifetime may be stabilized by cell-type-specific gene transcription, which eliminates the expression of unnecessary genes and transposable elements. Note that cell division leads to only partial rejuvenation, because each division introduces inevitable DNA replication errors, mitotic errors, and other forms of molecular damage[53, 62]. Moreover, the Hayflick limit imposes an additional constraint on how far this natural rejuvenation can extend in a normal (non-cancerous) somatic cell [34]. We argue that a full mechanistic understanding of aging and rejuvenation processes during interphase — including epigenetic remodeling of chromatin interactions [101, 49] — could inform tissue rejuvenation strategies that avoid errors associated with repeated cell division. An important caveat here is that it is still unknown whether architectural rescue alone is sufficient for tissue rejuvenation.

Another prominent manifestation of cellular aging is the loss of integrity of the nuclear lamina and the detachment of chromatin domains from the nuclear periphery. The nuclear lamina provides both mechanical stability for the nucleus and an organizing scaffold for chromatin. Lamina-associated domains (LADs) represent large heterochromatic genomic regions that interact with the lamina and contribute to spatial segregation of heterochromatin and euchromatin. The interactions of LADs with the lamina are identified by the DamID method and their nuclear position can be traced by microscopy [105, 103]. With aging – and more dramatically in premature aging disorders such as Hutchinson–Gilford progeria syndrome (HGPS) -— the structural coupling between the chromatin and lamina weakens. The resulting reorganization of chromatin is correlated with large-scale transcriptional dysregulation and changes in histone methylation patterns [69, 98]. In HGPS, mutations in the Lamin A (*LMNA*) gene lead to the production of progerin, a defective Lamin A isoform that compromises lamina integrity, linking nuclear envelope dysfunction directly to accelerated epigenetic aging.

Fruit flies (*Drosophila melanogaster*) have a short lifespan, a small genome, and extensive genetic tools, making them a useful and simpler model for studying aging [31]. Unlike mammals, which have three lamin genes (*LMNA*, LMNB1, LMNB2) and multiple splice variants, *Drosophila* has only two lamin genes (*LamC –* A-type lamin gene and *Dm0 –* B-type lamin gene), which makes fruit flies ideal for studying the connection between lamin proteins and aging. Multiple studies have demonstrated that both A-type and B-type Lamins in *Drosophila* can vary with age depending on tissue, and that such variation correlates with decline in tissue function, chromatin destabilization, or genomic instability[46, 55, 108, 13, 52, 28]. Experimental knock-down of *Dm0* in neurons led to a shorter lifespan, progressive motor impairment, and a loss of dopaminergic neurons in the protocerebral anterior medial cluster of the *D. melanogaster* brain [79]. The Lamin B mutation causes heterochromatin decompaction and transposon activation in the Drosophila germline, which is similar to what occurs during physiological aging [73]. This indicates a close link between heterochromatin maintenance at the nuclear periphery and aging mechanisms. The ectopic expression of Lamin B in dopaminergic neurons in the anterior medial region of the protocerebrum improves male flies’ locomotor activity but with little impact on their stress responses or lifespan [58]. Together, these studies indicate that Lamin depletion is a reasonable model of age-related changes in chromatin structure. The causal role of lamina–chromatin interactions in maintaining nuclear organization is well known [11], and has also been probed computationally[24]. In particular, recent models based on polymer physics [14, 102, 22] have demonstrated that disruption of lamina attachment sites can recapitulate experimentally observed chromatin remodeling that follows Lamin depletion or degradation [103, 9]. More directly in the context of aging, Brownian-dynamics simulations of a single human chromosome demonstrated that altered heterochromatin–lamina and heterochromatin–heterochromatin interactions can drive senescence- and progeria-like chromosome reorganization [14]. These models underscore the strong physical coupling between LADs and the nuclear envelope, suggesting that the 3D architecture of chromatin is not only biologically regulated but also mechanically stabilized by its interactions with the lamina[45].

A central unresolved question in epigenetic rejuvenation is whether the aged 3D architecture of the genome can, in practice, be returned to a youthful architectural state. If epigenetic age reversal is to restore normal cellular function, it is difficult to imagine a truly rejuvenated nucleus retaining an aged, disorganized 3D genome architecture. However, recovery of a complex spatial organization is not guaranteed. Even the refolding of medium-sized proteins can be slow, incomplete, or dependent on chaperones, despite the fact that the native fold is encoded by a single polymer sequence. The interphase genome is vastly larger, confined, topologically constrained, and organized only statistically rather than as a single fixed structure. There is an additional reason why such recovery is not guaranteed. The functional interphase architecture of large eukaryotic chromosomes is not generally expected to be an equilibrated polymer state. For example, chromosome territories were argued to likely arise as a kinetic consequence of decondensation from compact mitotic chromosomes, and estimated global equilibration times of about 5 years for *Drosophila* chromosomes and about 500 years for human chromosomes, far exceeding the relevant interphase timescales [91]. Subsequent polymer-physics and data-constrained 3D genome models have emphasized the same general picture: interphase chromosome organization reflects a combination of slow topological relaxation, chromosome confinement, sequence-dependent interactions, and chromosome–nuclear-envelope contacts, rather than rapid equilibration to a unique mixed state [42, 82, 26, 5]. Therefore, restoring the WT interaction landscape does not necessarily restore the WT interphase conformational ensemble: the biologically relevant state may be metastable[14], history-dependent[14], and constrained by the path taken out of mitosis.

Thus, an important physical question – and the main focus of this work – is whether age-associated disruption of chromatin architecture produces kinetically inaccessible states, or whether a young-like, far-from-equilibrium architectural ensemble can be recovered on biologically meaningful timescales when the appropriate organizing interactions are restored.

Here we test this idea in a coarse-grained, experimentally grounded model of the *Drosophila* interphase nucleus. In this model, age-associated architectural degradation is represented by weakening chromatin–lamina interactions, a perturbation motivated by the known relationship between lamina dysfunction, heterochromatin disorganization, and aging/progeroid phenotypes. We then ask whether restoring the youthful interaction landscape is *sufficient* to drive spontaneous recovery of the young-like chromatin ensemble.

An important technical step toward answering this question is the availability of a useful metric for aging-associated changes in 3D chromatin organization. Although epigenetic aging clocks based on DNA methylation patterns are widely used as age estimators[38, 76, 64, 70], they do not capture the full complexity of cellular aging. Ultimately, more comprehensive age prediction models should include the degree of deterioration of the 3D architecture of chromatin inside the cell nucleus. Recent studies suggest that at the higher organizational level, the nuclear structure and spatial organization of chromatin can be explored as new estimators of aging. For example, the morphology of cell nuclei has been studied as a biomarker of aging [81] and has been used to model cellular age with the help of an interpretable deep learning approach [83]. Other studies demonstrated that the 3D genome organization changes dramatically during the cellular aging process [101, 49], further supporting the idea that aging clocks that incorporate key aspects of chromatin architecture can be developed. Here, we take a step in this direction.

## 2 Methods

### 2.1 Entropy of Hi-C matrix

The concept of entropy has been used in the field of chromatin data analysis and simulation [81, 111, 87, 50]. Here we seek the simplest possible, yet physically meaningful, entropy-based metric that can be computed from a Hi-C contact matrix. Specifically, we introduce the notion of entropy $S\left(A_{ij}\right)$ of a contact (Hi-C) matrix $\left\{A_{ij}\right\}$ that describes contact frequencies between N structural elements (loci) i=1,2,…N of a chromatin (polymer) chain. Naturally, we assume $A_{ij}\geq 0$. Next, $A_{ij}$ needs to be converted into a probability distribution $P_{ij}$, to which the usual Shannon form, $S=-\sum_{ij}P_{ij}\;log\;P_{ij}$, can be applied, with all expected properties of S, such as that it is maximized by a uniform underlying probability distribution. There is more than one way to convert $A_{ij}$ into $P_{ij}$. Since our goal is to derive an expression for S that treats all “non-trivial” elements of the contact matrix on the same footing, as opposed to, e.g., focusing on near-diagonal elements [50], there are three intuitive ways to normalize $A_{ij}$: global normalization $P_{ij}^{\mathrm{global}\;}=A_{ij}/\sum_{ij}A_{ij}$, as well as row-based and column-based normalizations $P_{ij}^{\mathrm{row}\;}=A_{ij}/\sum_{k}A_{ik}$ and $P_{ij}^{\mathrm{col}\;}=A_{ij}/\sum_{k}A_{kj}$, respectively. Once the normalization is chosen, we can define

(1) $$S=-\sum_{ij}P_{ij}\log_{2}P_{ij},$$

where we have chosen $log_{2}$ to specify Shannon [97] entropy, as opposed to ln for Gibbs entropy [80], to avoid any confusion with the standard entropy of a polymer chain defined by counting conformational states consistent with macroscopic constraints[92]. The following limiting, albeit hypothetical, cases help choose the best normalization strategy and justify the definition, Eq. 1, specifically in the context of 3D organization of chromatin. On the one hand, a completely disorganized contact map would have the same $P_{ij}$ values for all its elements, which means that every locus has an equal probability of being in contact with every other. Presumably, this would be a *maximum-disorder* state [8] of chromatin of maximum entropy. On the other extreme is a purely diagonal matrix of contacts: $P_{ii}=1$, and $P_{ij}=0$ for all other elements outside the diagonal, i≠j. Physically, this contact matrix would describe chromatin without any interactions except between the neighboring loci along the chain. All real biological cases are in-between: the off-diagonal contacts $P_{ij}>0$. While the global normalization – a single normalizing constant for all the elements – has the appeal of being most intuitive, as well as of preserving the expected symmetry $A_{ij}=A_{ji}$ of the contact matrix, the global normalization has a fundamental drawback, as it violates entropy additivity for non-interacting systems. Namely, the entropy defined via globally normalized matrix is non-additive for independent, non-interacting subsystems, such as two independent, non-interacting chromosomes. Such systems are described by block-diagonal matrices, and one easily sees that the global normalization of the elements in each block depends on the presence and size of the other block(s), even if all the “cross” elements between the blocks are strictly zero. Fortunately, either the row-based or column-based normalization is free from this critical defect: the entropy of a block-diagonal matrix is strictly the sum of entropies of its blocks. Moreover, while the resulting probability matrices $P_{ij}$ are no longer symmetric, the symmetry is effectively restored as one takes the sum in Eq. 1: $S^{\mathrm{row}\;}=S^{\mathrm{col}\;}$. This is because, for a symmetric $A_{ij},P_{ij}^{\mathrm{row}\;}=P_{ji}^{\mathrm{col}\;}$, that is, the probability vector that defines a row in the row-normalized matrix is exactly the probability vector that defines the corresponding column in the column-normalized matrix, just transposed. We therefore choose the row-based normalization scheme for the rest of this work: $S=S^{\mathrm{row}\;}$. As defined, $S=\sum_{i}s_{i}^{\mathrm{row}}$, where $s_{i}^{\mathrm{row}\;}=-\sum_{j}P_{ij}^{\mathrm{row}\;}{log}_{2}P_{ij}^{\mathrm{row}\;}$; for the rest of this work we drop the label “row” for notational simplicity.

Note that with this definition, S grows proportionally to the total number of elements N; to facilitate comparison between contact matrices of different sizes in what follows, one can also consider the average entropy per row, S/N. In what follows, we will be excluding K near-diagonal elements, |i-j|≤K, for small K≥1 – the procedure yields an entropy measure that is sensitive to medium- and long-range structural features of chromatin, while removing trivial polymer connectivity effects and de-emphasizing very short range structural elements. Note that for a connected chain, $A_{ii}=1$ and $A_{i,i+1}=1$ always, so it makes sense to exclude these elements from the calculations to remove this trivial bias towards the diagonal; in practice, these elements are set to zero (masked) prior to the normalization procedure.

For simplicity and ease of interpretation, we use K=1 as the default, which removes self-contacts and immediate polymer-neighbor contacts while retaining near-local chromatin organization beyond trivial chain connectivity. The effect of larger near-diagonal masks (K>1) is examined in the SI. The central conclusions that both the lamins depletion and heat death increase contact-map entropy relative to WT, and that restoration of the WT interaction landscape returns the entropy toward WT values, are unchanged when K=4 is used. The more subtle relative ordering of the *lamins depleted* and *heat death* states is K-dependent, and is discussed in the SI.

Also note that, as expected, $S\left(A_{ij}\right)$ depends on the level of coarse-graining (resolution) of matrix $A_{ij}$ – direct comparison of entropy values obtained for different genomes or the same genome at different resolution should be avoided. A more subtle but related feature of $S\left(A_{ij}\right)$ is that $S\left(A_{ij}\right)$ depends on the averaging performed on $A_{ij}$: averages over more states or longer times, in the case of time-dependent $A_{ij}$, are expected to result in more “spread out” matrices, and hence higher $S\left(A_{ij}\right)$. Therefore, most meaningful comparisons should be limited to $A_{ij}$ averaged over the same time intervals and the same number of states/topologies sampled.

For chromatin structures made up of several distinct and unconnected chromosomes, because Hi-C matrices are block-diagonal at the chromosome level, we exclude (mask) short-range contacts |i-j|≤K only *within* each chromosome block, so that the exclusion does not affect contacts between different chromosomes. All practical details of extraction (in the case of Progeria Hi-C maps) and post-processing of Hi-C matrices, including normalization, exclusion of near-diagonal elements, entropy evaluation, and visualization are described in the corresponding sections of the SI.

### 2.2 Conformational Heterogeneity (C.H.) based on Hi-C contact probabilities

To quantify cell-to-cell heterogeneity in chromatin 3D organization, we adopt the concept of Conformational Heterogeneity, C.H., introduced in Ref. [67], where C.H. was originally defined as the standard deviation of average spatial Euclidean distances $\left\langle R_{s}^{\left(k\right)}\right\rangle$ between genomic loci separated by distance s over an ensemble of single cells [67]:

(2) $$C.H.(s)=\sqrt{\frac{1}{n}\sum_{k=1}^{n}{\left(\left\langle R_{s}^{\left(k\right)}\rangle -\langle R_{s}\right\rangle \right)}^{2}},$$

where $\left\langle R_{s}^{\left(k\right)}\right\rangle$ is the average spatial distance between all pairs of loci at distance s in the k-th conformation, and $\left\langle R_{s}\right\rangle$ is the average over all n conformations (cells).

In this work, we replace the mean physical distance $\left\langle R_{s}^{\left(k\right)}\right\rangle$ with the mean row-normalized Hi-C contact probability $\left\langle P_{s}^{\left(k\right)}\right\rangle$ for each sample, so that the C.H. can be computed directly from experimental Hi-C probabilities. The revised formula reads:

(3) $$C.H.\left(P_{s}\right)=\sqrt{\frac{1}{n}\sum_{k=1}^{n}{\left(\langle P_{s}^{\left(k\right)}\rangle -\left\langle P_{s}\right\rangle \right)}^{2}},$$

where $\left\langle P_{s}^{\left(k\right)}\right\rangle$ is the mean probability of contact at genomic distance s for the k-th sample (after row normalization and exclusion of near-diagonal contacts with |i-j|≤1), and $\left\langle P_{s}\right\rangle$ is the average value across all samples.

This formula yields a direct measure of C.H. based on the contact probabilities available from the Hi-C map, thus bypassing the need for a full 3D structure in Euclidean space, which can be difficult or impossible to obtain.

In this work, each of the five distinct states of chromatin: WT, *heat death*, *lamins depleted*, and both “rejuvenated” states is represented by sets of model nuclei, generated as described in the following section. Each set, which comprises multiple chromatin configurations, corresponds to one data point in Figs. 4 and 6. The (average) Hi-C map corresponding to each set is used as a data point in Eq. 3. In the implementation of Eq. 3 used in this work, both upper and lower matrix triangles are utilized after normalization.

### 2.3 Chromosome Intermingling

Inter-chromosomal intermingling is quantified as the fraction of the chromatin-occupied nuclear volume that is shared by two or more chromosome territories, by analogy with the cryo-FISH “intermingling volume” described in Ref. [10]. Here, each of the six major chromosome arms of the fruit fly (2L, 2R, 3L, 3R, 4 and X) is treated as a distinct territory.

For every conformational snapshot, the bounding box is discretized onto a regular cubic grid of spacing dx=0.1σ(σ= simulation length unit, which is 1 *μ*m in this work). Each monomer is assigned a spherical footprint of radius R=0.2σ: a voxel is marked as occupied by an arm if its center lies within R of any bead of that arm, and the union of such voxels defines the arm’s territory. A voxel is classified as *intermingled* when occupied by at least two of the six arms. The per snapshot (per frame) intermingling index is computed as

(4) $$I=\frac{N_{\geq 2}}{N_{\geq 1}},$$

where $N_{\geq 2}$ and $N_{\geq 1}$ are the numbers of voxels occupied by at least two and at least one arm, respectively; *i.e.* the fraction of the nuclear volume occupied by the chromatin in which territories overlap. By definition, I is dimensionless, bounded in [0, 1], and independent of the grid origin and of the σ→μm calibration. The intermingling index of fruit fly chromatin is shown in Figs. 5 and 7. Further details and a connection with Ref. [10] are available in the SI.

### 2.4 The dynamic model of the fruit fly nucleus

Our approach uses the well-established computational strategy [91, 54, 102, 59, 77, 51, 86, 102, 71, 93, 18, 44, 56, 15, 26, 23, 17] to represent chromatin at an appropriate coarse-grained level. Specifically, we employ a representation of the fruit fly genome at the level of topologically associating domains (TADs)[102]. Each TAD is modeled as an interacting polymeric unit whose effective attractions reflect the underlying chromatin state and affinity to the lamina. This level of abstraction captures both the essential large-scale organization of interphase chromatin and the stochastic thermal dynamics that underlie its remodeling. The model allows systematic variation of physical parameters—such as the strength of lamina–TAD interactions, inter-TAD contact potentials, and nuclear confinement—to reveal the energetic and entropic principles governing chromatin reorganization.

The 3D structures of fruit fly chromatin used in this work are generated using the model fully described in Ref. [102] and recently employed in, *e.g.*, Refs. [1, 67]. Briefly, the model describes the time evolution of embryonic *Drosophila* female interphase nuclei at spatial resolution of topologically associating domains (TADs), *i.e.* ~100 kb of chromosome length. In the model, a diploid set of four female chromosomes is represented by four chains of spherical beads. Each bead accounts for a pair of homologous TADs in paired homologous female chromosomes; the beads are of variable size, the average radius of the bead is 0.091 *μ*m. In addition to 1169 TAD-beads, the chromosome chains also contain the pericentromeric constitutive heterochromatin (HET) and centromeric (CEN) regions, represented by additional 6 and 4 beads, respectively, and an almost centrally located nucleolus. The chromosome chains and the nucleolus are confined to a spherical enclosure representing the nuclear envelope (NE), the inner surface of the nuclear envelope is lined by the nuclear lamina (NL) – a meshwork of Lamins. A supplemental movie is available that illustrates ~ 30 seconds of the time evolution of the model nucleus.

Four initial configurations of the chromosomes – mutual arrangement of the chromosome chains – are considered[102]. To account for the natural variability of nucleus size in different nuclei, 3 slightly different nucleus sizes (2 ± 0.05*μm*) are considered for each topology [102]. In this work, each nucleus is simulated using Langevin dynamics corresponding to three intervals of about 3 hrs. of biological time each.

The model agrees with experimental data on multiple independent metrics [102]: the Pearson’s correlation coefficient of the model-derived Hi-C map with the experimental wild type (WT) Hi-C map is 0.954; reasonably distinct chromosome territories 11 hrs into the interphase; model-derived radial chromatin density distribution is in good agreement with the experimental one. The model is also consistent with the experimentally observed stochasticity and cell-to-cell variability of the attachment of LADs to the NE.

The interaction parameters of the models were tuned to reproduce the TAD-NE contact probability data [84] and the coarse-grained TAD-TAD contact probability matrix, derived by Li et al. [54] from the experimental bulk Hi-C map [95]. Each TAD-related bead in the models represents two paired homologous TADs with approximately 100 kb of DNA in each, resulting in one polymer chain for each of two paired homologous chromosomes in the diploid female *Drosophila* genome. These homologous TADs and chromosome pairing are observed experimentally [29, 19, 2, 16], and explained within a theoretical model [16]. As in our previous work, the entire simulated ensemble consisted of 18 model nuclei representing 6 properly weighted chromosomal arrangements in 3 replicas to account for 3 different nucleus sizes used [102]. Langevin dynamics are used to simulate 18 distinct nuclei and generate 18 trajectories, each representing hours of the biological time evolution of the interphase chromatin, *e.g.*, Figs. 4 and 6. The model fully accounts for the different experimentally observed topologies (mutual arrangements of the chromosomes [36]) of the *Drosophila* chromatin. The relation between the experimental and simulation timescales was previously determined by comparing the calculated and experimental mean square displacement (MSD) of a chromosomal locus as a function of time; see Ref. [102] for details. The simulations took approximately 10 days on 18 computing cores. Unless otherwise specified, all of the model-derived quantities, including the bulk Hi-C maps $A_{ij}$, the entropy $S\left(A_{ij}\right)$, and the conformational heterogeneity C.H., are calculated as time averages, averaged over all 18 trajectories generated by the model. Each data point in Figs. 4 and 6 is an average over 10 minutes, or 6065 conformational snapshots, except for the inset Hi-C maps, which are averages over 3 hrs, or 109182 snapshots. For the computation of C.H., Fig. 8 and Table 2, each state of chromatin, *e.g.*, WT, *heat death*, etc. is represented by its corresponding Hi-C map averaged over the same snapshots as described above. The average intermingling, ⟨I⟩, is computed over 1000 snapshots representing each state of chromatin (3 hrs of simulation), see SI.

#### 2.4.1 *lamins depleted* model nuclei.

Lamin depletion is modeled by setting the affinity of LAD-containing TADs for the NE to zero, see Ref. [102] for details. In particular, it was shown previously [102] that the model reproduces well the changes in chromatin distribution induced by Lamin depletion. The selection of the representative ensemble is the same as for the wild type (WT) nuclei described above. We refer to this model state as *lamins depleted*.

#### 2.4.2 Hypothetical heat-death model nucleus

In this hypothetical nucleus, all of the attractive interactions are switched off, including LAD-NE (as in the *lamins depleted* state), all TAD-TAD, and the attraction to the nucleolus. The model is therefore reduced to self-avoiding chain(s) under confinement. The selection of the representative ensemble is the same as for the wild type (WT) nuclei described above.

### 2.5 Software used

VMD [40] was used to visualize chromosome models, *gnuplot* to make graphs, Juicebox[90] to visualize and pre-process Hi-C maps. ChatGPT v. 4 and 5 was used to generate and analyze FJC ensembles, explore various options for Hi-C entropy normalization in the simplified cases, and assist with grammar and style editing. The movies were generated with the assistance of Claude Code (Anthropic), using the Claude Opus 4.8 model. The authors assume full responsibility for the content of this manuscript.

## 3 Results

Using a realistic model of 3D chromatin organization in fruit fly, we test whether lamina-dependent architectural disruption that mimics age-associated chromatin disorganization can be reversed by restoring the interaction landscape characteristic of the WT nucleus, despite the complexity and highly dynamic nature of chromatin organization. To follow this process quantitatively, we use three complementary descriptors of 3D genome organization: Hi-C contact-map entropy introduced in this work, chromosome intermingling, and conformational heterogeneity. The relevant methodological details of the last two of these metrics, which have been previously introduced[67, 10], are presented in “Methods”. We begin by introducing Hi-C contact-map entropy and show that it traces the expected deterioration of 3D chromatin architecture, and is relevant to age-related changes in the 3D organization of chromosomes.

### 3.1 Shannon entropy of Hi-C contact map as a metric of chromatin disorder in the interphase

In what follows, we approximate the 3D chromatin organization by its corresponding contact probability matrix $P_{ij}$, which, in turn, can be approximated from a suitably normalized Hi-C map. From the examples discussed in the introduction, one can generally conclude that, at least for a single chromosome, the contact matrix becomes more “disordered” with age, or with the onset of conditions, such as Progeria, that can be considered as models of aging. Specifically, one expects that the “older” contact maps will have more distant off-diagonal contacts [101] compared to the younger nuclei, at least in the interphase, which is the focus of this work. To capture this type of disorder in a Hi-C matrix $A_{ij}$, we propose to use the familiar concept of entropy:

(5) $$S\left(A_{ij}\right)=-\sum_{ij}P_{ij}{log}_{2}P_{ij}$$

where we have chosen ${log}_{2}()$ to specify Shannon entropy [97], as opposed to ln() for Gibbs entropy [80] standard in Physics, for reasons mentioned in Methods. All important technical details, including the choice of normalization condition needed to convert the experimental Hi-C matrix to the matrix of contact probabilities $P_{ij}$, are presented in Methods.

**Figure 1:**
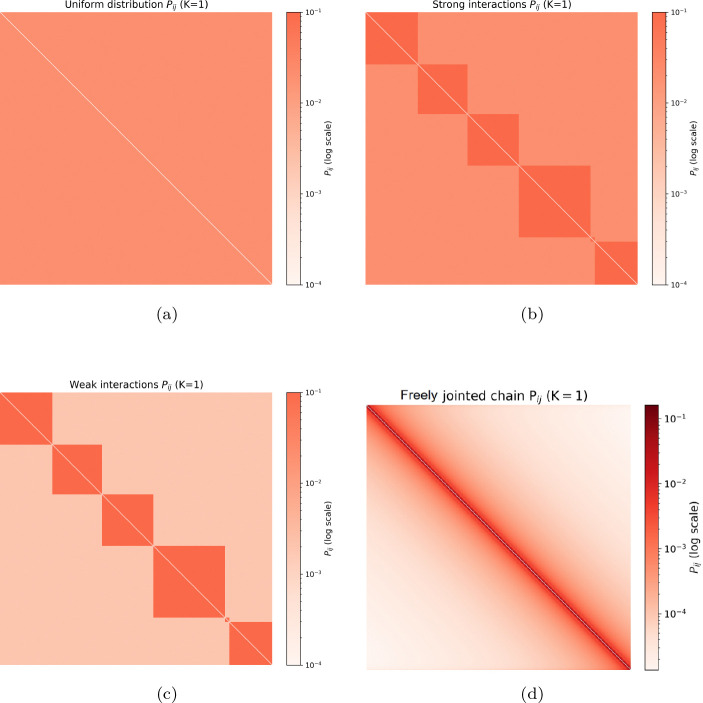
Examples of contact probability (Hi-C) square N×N symmetric matrices, N=1169, corresponding to four idealized systems. (a) A *maximum-disorder* state – equal probability of all contacts; (b) Strong interaction between different ”chromosomes” $P_{I}/P_{B}=10^{-1}$; (c) Weak interaction between different ”chromosomes” $P_{I}/P_{B}=10^{-2}$; (d) A single freely jointed chain of N beads.

The behavior of the proposed definition of Hi-C entropy is illustrated on several simplified and limiting cases, going from completely hypothetical to more biologically realistic, Fig. 1. First, we consider a completely uniform distribution of contacts $A_{ij}=const$, Fig. 1(a), corresponding to the completely hypothetical case of a *maximum-disorder* state, in which every locus has an equal chance to come in contact with any other locus, regardless of the inter-loci distance. Next, we consider a more realistic case of two block-diagonal matrices with added non-zero elements between the blocks, corresponding to several chromatin chains that interact, Figs. 1(b,c). For relevance to this work, the number of blocks and the length of each block are set equal to the number of chromosomes and the number of TADs in each chromosome of *D. melanogaster*, respectively, summing to the same total number of TADs [95, 102], see “Methods”. For simplicity, we assume uniform distributions of contact probabilities within each block $P_{B}$, and between blocks $P_{I}$, and vary their ratio $P_{i}/P_{B}$. Finally, in Fig. 1 (d), we consider a model of a single chromosome, whose pairwise contact matrix is that of a single freely jointed chain (FJC) of the same length as the entire genome of fruit fly, at TAD resolution, see “Methods” for details. In all cases, the total number of matrix elements $N\times N=1169^{2}$ is the same; the corresponding entropy values are summarized in Table 1, to which we have also added the trivial case of a completely diagonal contact matrix for which S=0.

As expected, the proposed definition of Hi-C map entropy anti-correlates with the amount of “structure” in the Hi-C map, with the highest entropy corresponding, as expected, to a completely uniform distribution. In the SI a brief exploration of potential applicability of the proposed Hi-C entropy $S\left(A_{ij}\right)$ in the context of mammalian cell aging during interphase is presented.

#### 3.1.1 Hi-C entropy increases in premature aging (Progeria) associated with lamina dysfunction

As discussed in the Introduction, lamins depletion is a reasonable model of age-related changes in chromatin structure. Therefore, the ability of the entropy-based metric to capture the corresponding increase in the Hi-C matrix disorder is critical.

**Figure 2:**
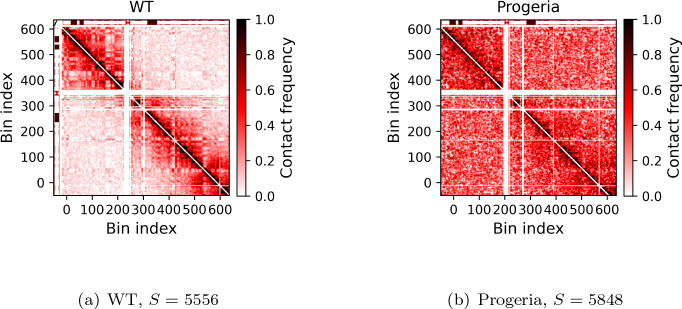
Row-normalized Hi-C contact probability maps for WT (Father-p18) and Progeria/HGPS (HGPS-p19) chromosome 7 fibroblast samples from Ref. [69]. The HGPS map is visibly more dispersed and less compartmentalized than the WT map and yields a higher Hi-C contact-map entropy, consistent with increased 3D genome disorder associated with lamina dysfunction in premature aging. The Hi-C entropy values are estimated from the published contact maps as described in the SI.

As an initial semi-quantitative consistency check, we compared WT and Progeria-associated Hi-C maps, Fig. 2. Hutchinson-Gilford Progeria syndrome (HGPS) is a premature aging disease that is often caused by a point mutation at position 1824 in LMNA. In-vivo, these changes can lead to loss of spatial chromatin compartmentalization and mis-regulation of transcription[69]. What is most relevant to us here is that this mutation disrupts heterochromatin-lamina interactions: similar *lamins depleted* states can be realized in fruit fly, both experimentally[103, 9] and computationally[102, 1, 67, 45].

As is evident from Fig. 2, the HGPS Hi-C map is visually more dispersed and less compartmentalized than the WT map, consistent with lamina-associated disruption of 3D genome organization. The Hi-C contact-map entropy is higher in HGPS than in WT, S=5848 versus 5556 bits, respectively. Thus, this comparison supports the expected direction of the entropy change in a progeroid context, whereas the Drosophila lamin-depletion data below provide a more directly relevant experimental comparison for the present model.

#### 3.1.2 Entropy of the Hi-C map increases with lamins depletion in fruit fly

**Figure 3:**
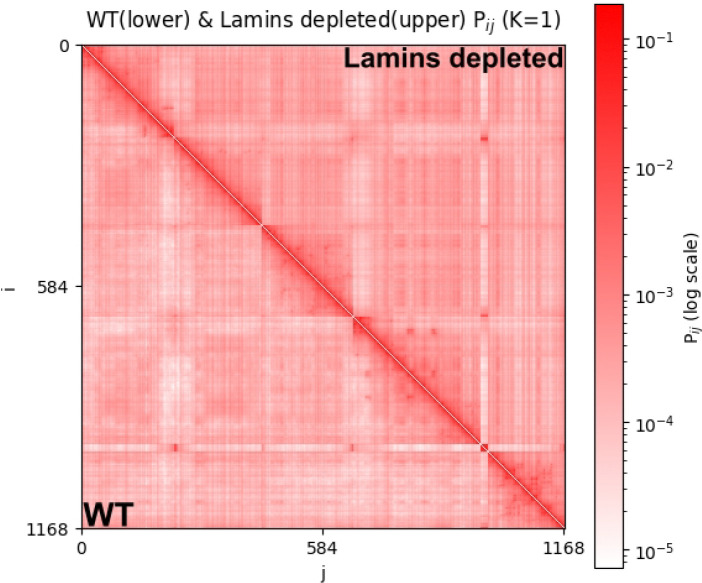
Changes in fruit fly chromatin architecture upon *lamins depleted*. Shown are WT (lower triangle) vs. *lamins depleted* (upper triangle) row-normalized contact probability map of the whole fruit fly nucleus at TAD resolution calculated directly from the experimental Hi-C data in Ref. [103]. The corresponding Hi-C entropy values are: $S_{WT}=8022$ bits, $S_{LM}=8849$ bits. The TAD index, which grows monotonically with the genomic coordinate, is shown along the axes. The average TAD size is about 100 kb; the specific partition of the fruit fly genome into TADs is described in Refs.[54, 102]

Experimental and computational works show that lamins depletions at the nuclear envelope (NE) in fruit fly can lead to significant re-arrangements of chromatin, as evidenced by major shifts in the chromatin density distribution upon lamins depletion [9, 102]. However, the corresponding changes in the Hi-C map are much more subtle, see, e.g., Ref.[102], and Fig. 3. Therefore, it is reassuring that the proposed metric, $S\left(A_{ij}\right)$, Eq. 5, is able to reliably capture the subtle increase in the contact map disorder induced by experimental lamins depletion (lamin knockdown[103]). We stress that this specific data point applies to the whole interphase nucleus of fruit fly, which makes it most relevant for this work. This result supports the usefulness of Hi-C contact-map entropy as a metric for detecting lamina-dependent, aging-associated changes in chromatin organization at the scale of the fruit fly nucleus.

Another critical observation is that the values of Hi-C entropy of the *experimental* WT and *lamins depleted* states, Fig. 3, are very close (within 1.6 %) to those calculated from Hi-C entropy maps of the WT and *lamins depleted* states predicted by our computational model of fruit fly chromatin, see also the following section for the specific numbers. This close agreement with the corresponding experiment provides support for the computational model’s ability to capture the direction and magnitude of lamina-depletion-induced changes in Hi-C entropy.

### 3.2 Restoration of a young/WT-like 3D state of chromatin in fruit fly

In this section, we address the key question of this work: can one reverse aging-associated disruption of the structural state of WT chromatin in fruit fly? In particular, to what extent can the youthful conformational ensemble of the chromatin be restored by eliminating/reversing the external factor or condition that has caused the deterioration of the original 3D structure of chromatin?

#### 3.2.1 Recovery from *lamins depleted* state

**Figure 4:**
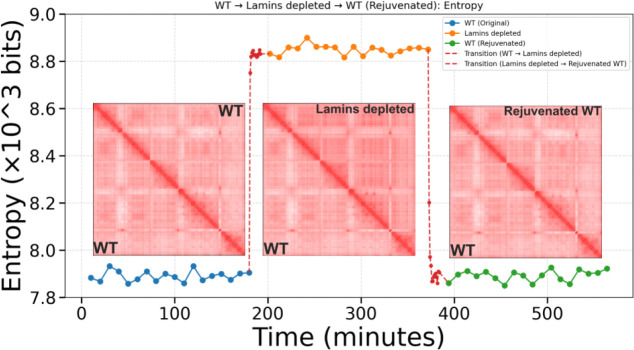
Transition from the WT to *lamins depleted* state of fruit fly is followed by its “rejuvenation” that fully restores the attractive interactions between LADs and the nuclear envelope (NE), present in the WT state and absent from the *lamins depleted* state. The transition to/from the *lamins depleted* state is initiated by an instantaneous switching off/on of the LAD-NE attractions, see Methods. The corresponding Shannon entropy values, averaged over time, are $\left\langle S_{WT}\right\rangle =7889\pm 5$ bits, $\left\langle S_{\mathrm{Lamins}\;\mathrm{depleted}}\right\rangle =8850\pm 6$ bits, and $\left\langle S_{\mathrm{Rejuvenated}\;\mathrm{WT}}\right\rangle =7893\pm 6$ bits, where the error bars represent the standard error of the mean. Each of the three Hi-C maps shown as the insets is a bulk Hi-C map averaged over the full 3-hour window corresponding to each of the three states: WT, *lamins depleted*, and rejuvenated WT. To facilitate visual comparison, the lower triangle block of each matrix is the same original WT, while the upper triangle shows the state corresponding to the time interval.

The key result of this work is summarized in Fig. 4, which shows the temporal evolution of the chromatin organization of the fruit fly nucleus as it transitions from the WT to the *lamins depleted* state, and then “rejuvenated” to the WT state. Specifically, after spending 3 hrs in its WT state, the chromatin undergoes an induced transition to *lamins depleted* state, in which all the attractive interactions between LADs and the nuclear envelope have been switched off, see Methods. As expected, the transition causes the entropy of the corresponding contact matrix to increase substantially. Remarkably, the values of Hi-C entropy maps of the *computed* WT and *lamins depleted* states, Fig. 4, are within 1.6 % of the *experimental* WT and *lamins depleted* states, Fig. 3. The corresponding changes in Hi-C entropy upon lamins depletion, S(laminsdepleted)-S(WT), are also very close between the simulated and experimental Hi-C maps. This agreement with the experiment further supports the use of our computational approach for generating experimentally testable predictions about lamina-dependent architectural recovery.

**Figure 5:**
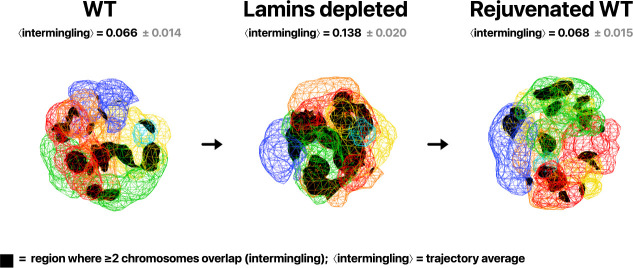
Chromosome territories along the WT → *lamins depleted* → WT rejuvenation pathway. The intermingling between chromosomes increases following transition to the *lamins depleted* state, and returns to its WT value upon the restoration of the attractive interactions between LADs and the nuclear envelope (NE), present in the WT state and absent from the *lamins depleted* state. Shown are example snapshots from each of the three states in Fig. 4; see also SI movies that show these states in dynamics. Each snapshot is selected so that its chromosome intermingling value (shown above the snapshot) corresponds to the trajectory average of the corresponding state. The intermingling regions are colored black. Each chromosome arm is colored differently, the gray sphere is the nucleolus. For clarity, only one out of four experimentally observed mutual arrangements (topologies) of the chromosomes (CYSX-6S nucleus topology[102]) is shown.

The deterioration of the chromatin organization in the *lamins depleted* state (upper triangle) is visible in the Hi-C map by comparing it with the WT map (lower triangle). That deterioration of the 3D organization of chromatin also manifests itself in substantial decay of chromosome territories[45], as evidenced by a two-fold increase in chromosome intermingling, Fig. 5. After equilibrating for 3 hrs in the *lamins depleted* state, the “rejuvenation” process is initiated: the attractions between LADs and the nuclear envelope, characteristic of the WT chromatin, are fully restored. Remarkably, the entropy of the resulting rejuvenated state quickly returns to its WT value within statistical uncertainty, see the caption of Fig. 4. The recovered state is nearly indistinguishable from the original WT state by bulk Hi-C contact-map similarity, see the insets in Fig. 4; the Pearson correlation coefficient between the original WT and recovered maps is 0.999902. Indeed, the difference map (not shown) between the two consists of matrix elements of the order of 10^−4^ or smaller. Consistent with near-restoration of the WT-like Hi-C map in the rejuvenated state of chromatin, chromosome intermingling is reduced to its WT level, Fig. 5, reversing the partial decay of chromosome territories induced by the lamins depletion. We stress that the restoration of the WT state refers to the corresponding statistical ensemble, and not to its individual conformations, which are highly dynamic on the relevant time scales[102] (see also the supplemental movie).

Thus, to the extent that Lamin depletion mimics an age-associated deterioration of chromatin architecture in fruit fly, the model predicts that this deterioration can be reversed by restoring the LAD–nuclear-envelope interactions characteristic of the young WT nucleus^a^. Given the complexity and dynamic nature of interphase chromatin, this recovery is non-trivial: the model does not impose the positions of individual chromatin domains, but instead restores the physical interaction landscape that stabilizes the young architectural ensemble. The test is therefore not whether the WT state is thermodynamically favored after restoration of the WT parameters; rather, the test is whether the disrupted chromatin ensemble can return to a WT-like ensemble on biologically relevant interphase timescales, without becoming kinetically trapped in an aged or disorganized architectural state. Because the same LAD–NE interaction term that was removed in the model is restored in the recovery simulation, the result motivates an experimentally direct test based on rescue of productive LAD–lamina attachment. Note that the transition times to and from the *lamins depleted* state are on the order of several minutes, negligible compared to the duration of the interphase, which can last up to 16 hours for fruit fly somatic cells [106, 89]. Although the correspondence to biological time used here[102] cannot be considered exact, it is nevertheless expected to be accurate to roughly an order of magnitude, implying that upon restoration of the WT LAD-NE attractions, the WT state of chromatin is restored relatively fast.

The central experimentally testable prediction is that restoration of functional LAD–lamina interactions may be sufficient, under otherwise permissive conditions, to drive recovery toward a young/WT-like 3D chromatin architecture after lamina-dependent disruption. Importantly, the predicted intervention is not merely increased lamin abundance, but restoration of productive LAD association with the nuclear envelope. This distinction is essential experimentally: lamin expression and nuclear-envelope localization should be verified, but successful rescue of the model parameter should be assessed directly by LAD–lamina readouts such as DamID and/or fluorescence in situ hybridization (FISH)-based measurements of LAD radial positioning. If the model is correct, inducible lamin depletion followed by inducible lamin restoration should produce a reversible architectural trajectory: increased Hi-C map entropy, weakened chromosome territories, and increased conformational heterogeneity after depletion, followed by a return to WT values after LAD–lamina contacts are restored. One practical route to test this prediction is as follows. The restoration of a youthful state of chromatin can be achieved by using GAL80(ts) or GeneSwitch-GAL4 inducible systems [6, 75] to express lamin in fruit fly cells. Lamin repression or depletion can be performed using the UAS-RNAi-lamin construct, while restoration of lamin expression can be done using the UAS-lamin construct. To ensure robustness, lamin expression should be manipulated using both the RU486-inducible GeneSwitch-GAL4 and the temperature-sensitive GAL80(ts) systems. The key experimental requirement is to verify that the intervention restores LAD–lamina association, not only lamin protein abundance. If such experiments are performed, it will be important to determine how LADs reorganize spatially in response to these perturbations using FISH or DamID [105, 103]. In parallel, Hi-C data must be obtained to determine whether chromatin contact maps, Hi-C map entropy, chromosome-territory organization, and cell-to-cell conformational heterogeneity return toward their WT values after lamin restoration.

Finally, if restoration of young-like 3D genome architecture contributes to cellular or organismal rejuvenation, these simulations motivate testing of whether restoration of functional LAD–lamina interactions in lamins depleted or aged flies improves aging-associated phenotypes, including Drosophila lifespan. Experimental studies have revealed a direct link between the function of lamins and organismal aging. *Drosophila* LamC mutations modeled after human progeroid LMNA mutations result in premature aging of adult flight muscles, including decreased levels of specific mitochondrial RNA transcripts and progressive mitochondrial degradation [55]. Knock-down of Dm0 in neurons led to a shorter lifespan, progressive impairment, and a loss of dopaminergic neurons in the protocerebral anterior medial cluster of the *D. melanogaster* brain [79]. The hypothesis that lamin restoration may improve lifespan or other aging-associated phenotypes could be tested by restoring WT lamin to physiologically normal levels, while also verifying recovery of functional LAD–lamina attachment. To be more precise, our model predicts that restoring functional LAD–nuclear envelope attachments — not merely lamin abundance — is sufficient to recover a WT-like 3D chromatin ensemble. In the case of rejuvenation of aged fruit fly, resetting the ”young” epigenetic states of the LADs may be necessary as well to make LADs functional. For example, the aged-to-young cells ratio of the H3K27ac signal, a key marker for active enhancers and promoters, was inversely correlated with the basal H3K27ac signals, which means that regions that were strongly acetylated in young cells tended to lose H3K27ac with age, and vice versa. The observed blurring of the originally sharp H3K27ac pattern in dividing mouse embryonic fibroblasts demonstrates that the aging process corrupts epigenetic information [110]. Upon experimental lamina disruption in the *Drosophila* S2 cell line, the LADs become more acetylated at histone H3 and less compact after cells complete mitotic divisions [103]. The relevant epigenetic marks accumulate mainly during the G1 phase, remain relatively stable during the same interphase, and are removed from chromatin during mitosis [66]. However, epigenetic marks may also deteriorate in non-dividing cells during aging. For example, one study has shown increased chromatin accessibility and the loss of the heterochromatin mark H3K9me3 in the non-dividing excitatory neurons in the old mice compared to young ones [113]. In *Drosophila*, both H3K4me3 and H3K36me3 globally decrease across all actively expressed genes in non-dividing aging photoreceptors [41]. The senescence of *Drosophila* postmitotic fat body cells involves a reduction in Lamin B associated with age, which contributes to the loss of heterochromatin and the derepression of genes involved in immune responses [13]. Therefore, if some components of epigenetic aging accumulate during interphase, then partial architectural rejuvenation may be experimentally achievable in non-dividing cells. This highlights a knowledge gap in the direct mapping of epigenetic changes within LADs during normal aging in non-dividing cells, such as neurons. Studies of neuronal aging show lamina weakening, compartment shifts, and heterochromatin erosion [49, 112], but the analysis of histone and DNA methylation has not yet been conducted.

#### 3.2.2 Recovery from a limiting case ” *heat death* ” state

Encouraged by the model prediction that lamina-depletion-induced architectural changes can be largely reversed by restoring WT-like LAD–NE interactions, we next test the recoverability of the chromatin architecture in an extreme limiting case. This stress test is designed to explore the extent of disorganization of a 3D genome architecture that can still be reversed on timescales much shorter than the duration of interphase. To this end, we consider a hypothetical fruit fly nucleus in which both all LAD–NE attractive interactions and all TAD–TAD attractive interactions have been switched off, see Methods. The resulting state of fruit fly chromatin, which we dub ”*heat death* ” for notational simplicity, is represented by four separate self-avoiding polymer chains under confinement (the two chromosomes 2 and 3, plus the two chains corresponding to chromosome “X” and “4” confined within the NE).^b^

**Figure 6:**
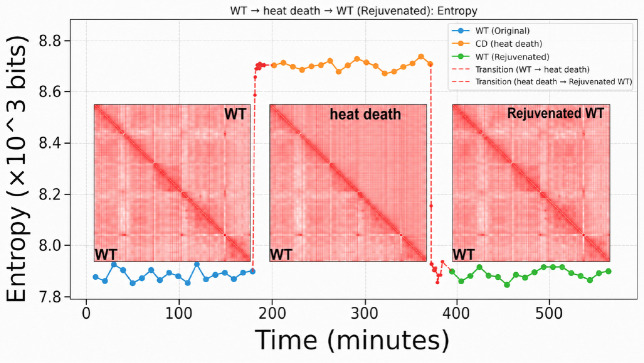
Transition from the WT to “*heat death* ” state of fruit fly is followed by its “rejuvenation” that fully restores the attractive interactions between all the elements of chromatin, including TADs, as well as between LADs and the nuclear envelope (NE), appropriate for the WT state and absent from the “*heat death* ” state. The transition to/from the “*heat death* ” state is initiated by an instantaneous switching off/on of all of the attractive interactions, see Methods. The corresponding Shannon entropy values, averaged over time, are $\left\langle S_{WT}\right\rangle =7890\pm 5$ bits, $\left\langle S_{\mathrm{heat}\;\mathrm{death}}\right\rangle =8699\pm 4$ bits, and $\left\langle S_{\mathrm{Rejuvenated}\;\mathrm{WT}}\right\rangle =7895\pm 5$ bits, where the error bars represent the standard error of the mean. Each of the three Hi-C maps shown as the insets is a bulk Hi-C map averaged over the full 3-hour window corresponding to each of the three states: WT, “*heat death* ”, or rejuvenated WT. To facilitate visual comparison, the lower triangle block of each matrix is the same original WT, while the upper triangle shows the state corresponding to the time interval.

**Figure 7:**
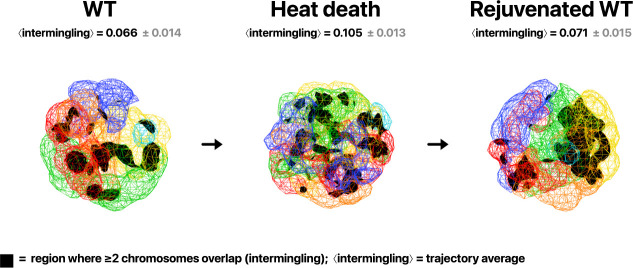
Chromosome territories along the WT → *heat death* → WT rejuvenation pathway. The intermingling between chromosomes increases following transition to the *heat death* state, and returns to its WT value upon the restoration of the attractive interactions between the TADs, and between LADs and the nuclear envelope (NE), present in the WT state and absent from the *heat death* state. Shown are example snapshots from each of the three states in Fig. 6; see also SI movies that show these states in dynamics. Each snapshot is selected so that its chromosome intermingling value (shown above the snapshot) corresponds to the trajectory average of the corresponding state. The intermingling regions are colored black. Each chromosome arm is colored differently, the gray sphere is the nucleolus. For clarity, only one out of four experimentally observed mutual arrangements (topologies) of the chromosomes (CYSX-6S nucleus topology[102]) is shown.

As before, we begin our simulation with 3 hrs in the WT state, Fig. 6, followed by an instantaneous switch off of all biologically relevant, and carefully tuned [102] attractive interactions within the entire chromatin at the TAD resolution, which yields a strongly simplified *heat death* state dominated by polymer connectivity, confinement, and excluded-volume effects. Apart from near-diagonal contacts arising from chain connectivity, the contact map loses much of the WT-specific long-range structure, see the upper triangle of the middle Hi-C map inset in Fig. 6. It is therefore particularly noteworthy that the original WT state of the chromatin is essentially restored within several minutes upon restoring the relevant interactions within the chromatin and with the NE, as judged by contact-map entropy, Hi-C map similarity, and chromosome-territory intermingling. The Pearson correlation coefficient between the Hi-C maps of the original WT and the “rejuvenated after *heat death* ” states is 0.999987; the difference map (not shown) between the two has matrix elements on the order of 10^−4^ or smaller. Consistent with near-restoration of the WT-like Hi-C map, chromosome intermingling is reduced to approximately the WT level, Fig. 7, reversing the *heat death* -induced increase in territory overlap. The subtle entropy ordering between *lamins depleted* and *heat death* states is discussed in the SI.

The above results indicate that the WT-like 3D architecture can be recovered not only from the *lamins depleted* state, but also from a more severely perturbed model state, *heat death*, in which all attractive interactions have been switched off.

Within the computational model, the “rejuvenation” is achieved by switching back on the inter-loci (TAD-TAD) and chromatin-lamina interactions specific to the young nucleus. Can this prediction be tested experimentally? In fruit flies and mosquitoes, evolutionarily conserved chromatin interactions can occur between genomic loci that are separated by several megabases [72, 65]. It was shown that CTCF (CCCTC-binding factor) and GAF (GAGA-associated factor) play direct roles in higher-order meta-domain interactions in *Drosophila* [72]. Further studies demonstrated that some specific long-range chromatin interactions depend on CTCF or GAF and Vostok transcription factors [72, 74, 39]. TADs arise mainly from cohesin-mediated loop extrusion and CTCF-defined boundaries in mammals [3, 88] or from chromatin-state partitioning, active promoters/transcription, diverse architectural proteins and by boundary pairing in fruit flies [109, 7, 68]. Disruption of TADs would also disrupt TAD-TAD interactions. Therefore, in order to abolish much of the attractive TAD–TAD interaction landscape across the genome, the core architectural components should be the target in the experiment. It has been shown that loss of cohesin eliminates most TADs and cohesin/CTCF loops and relaxes domain architecture in mammalian cells [3, 88]. This flattens intra-TAD structure globally and reduces structured TAD–TAD interaction patterns, although some compartmental or Polycomb-driven contacts persist [88]. Thus, to experimentally abolish most native attractive TAD–TAD interactions at genome scale, the most direct validated strategy is cohesin depletion, which dismantles loop-extrusion–based domains. Such experiments have not yet been performed in combination with lamina depletion. If cells with broadly disrupted architectural interactions remain viable, future experiments could in principle test whether partial restoration of selected inter-loci and chromatin–lamina interactions promotes architectural recovery, although such experiments would be technically demanding.

#### 3.2.3 Conformational heterogeneity of chromatin across cells increases, revesibly, with lamins depletion

It was previously hypothesized[102] that the removal of attractive LAD-NE interactions, which stabilize overall chromatin organization, can lead to increased heterogeneity of chromatin conformations. Here we test this hypothesis, and explore whether the trend can be reversed by restoring the WT LAD-NE attractions. To this end, we employ a newly developed [67] quantitative metric of cell-to-cell *conformational heterogeneity*, C.H., of chromatin organization in 3D. By construction, C.H.(s) averages out conformational variability within each given nucleus, thereby focusing specifically on cell-to-cell variability. That is, C.H.(s) would yield exactly zero for a hypothetical case of identical nuclei, even if the chromatin conformations within each nucleus were highly variable. Here, we adapt the metric so that it can be computed directly from the HiC map, which has multiple practical advantages, see “Methods”. Specifically, C.H. at genomic separation s is defined as the standard deviation of the average probability of contact between chromatin loci separated by genomic distance s.

To summarize the overall heterogeneity by a single number per chromatin state, we average $C.H.\left(P_{s}\right)$ over all available genomic separations s on the *whole* nucleus, Table 2. This provides a compact, global measure of conformational variability that can be directly compared across nuclear states and with the entropy-based analysis presented above.

**Figure 8:**
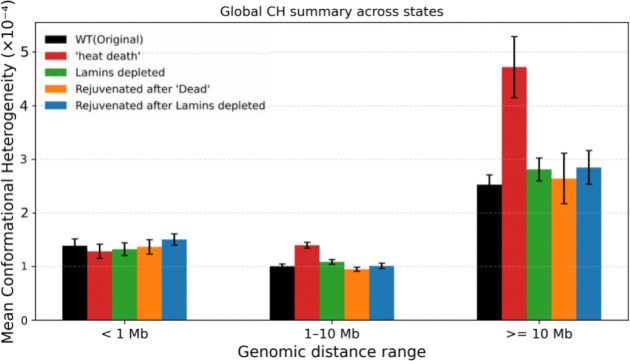
Whole-genome comparison of conformational heterogeneity (C.H.) across nuclear states. For each Hi-C map, the contact probability matrix is row-normalized after excluding the diagonal. C.H. is defined as the standard deviation of P(s) across replicate maps for each state. The bars report C.H. averaged within three genomic distance ranges (< 1 Mb, 1–10 Mb, and ≥ 10 Mb). Error bars indicate jackknife standard errors across replicate Hi-C maps.

Across the whole nucleus, conformational heterogeneity increases after severe chromatin disruption (*heat death*) and after the less drastic lamin-depletion perturbation, while restoration of interactions returns the system toward WT levels. Chromosome-arm–resolved C.H.(s) values, see the SI, support the whole-genome conclusion in Fig. 8 and Table 2. These show that, while the magnitude and detailed distance dependence of C.H.(s) vary across individual chromosome arms, the relative ordering of nuclear states is broadly consistent with the whole-genome summary. These arm-specific results further support the use of a global C.H. measure in the main text, while highlighting heterogeneity in the contributions of different chromosomal regions.

In summary, we suggest that cell-to-cell conformational heterogeneity of chromatin increases when either chromatin–NE interactions alone or both chromatin–NE and TAD–TAD interactions are disrupted. Thus, the substantial loss or dysfunction of lamins during aging or disease [11, 58, 69] may contribute to increased disorder in gene expression by enhancing variability in global chromatin architecture. These findings highlight a key role of chromatin–NE and TAD–TAD interactions in the establishment of non-random three-dimensional chromatin organization and in the preservation of its integrity against the inherent cell-to-cell variability of chromatin conformations. Importantly, our simulations further suggest that the increase in conformational heterogeneity associated with disruption of chromatin–NE interactions (and even in the extreme case of disruption of both chromatin–NE and TAD–TAD interactions) can be mostly reversed by restoring these interactions to WT values. We propose that the predicted changes in cell-to-cell conformational heterogeneity can be tested experimentally using single-cell Hi-C or related single-cell 3D genome assays[104]. We further suggest that the proposed association between cell-to-cell heterogeneity of 3D genome architecture and cell age is worth exploring in more detail.

## 4 Conclusions

The main motivation for this work was to explore the extent to which disorganization of chromatin conformation — a potentially important component of age-related deterioration of the epigenetic landscape — can be reversed by biologically meaningful manipulations, on timescales much shorter than interphase. Given the extraordinary complexity of hierarchical chromatin compaction in the interphase nucleus, our initial expectation was that even a partial restoration would be difficult.

The central conclusion and main testable prediction of this work is that the disorganization of the spatial state of fruit fly chromatin induced by lamins depletion, which mimics age-related changes, can be reversed to near-WT values, by the measured architectural metrics, when WT-like LAD–nuclear-envelope interactions are restored.

The recovery observed here should not be interpreted as a trivial consequence of restoring the same LAD–NE interaction term that was removed during lamins depletion. If interphase chromatin were an equilibrated polymer ensemble, then restoring the WT interaction landscape would be expected to eventually restore the WT equilibrium distribution. However, large-scale interphase chromosome organization is widely understood to be far from equilibrium on cell-cycle timescales. In this context, the nontrivial result is that restoring WT-like LAD–NE interactions returns the disrupted chromatin ensemble to a WT-like architectural state on interphase timescales, without imposing individual TAD positions or chromosome territories.

The most direct biological prediction of this work is that restoration of functional LAD–lamina interactions, rather than lamin abundance alone, should promote recovery toward a young/WT-like 3D chromatin architecture after lamina-dependent disruption. This prediction can be tested by inducible lamin depletion and restoration in *Drosophila*, with DamID or FISH used to verify recovery of LAD–lamina association and Hi-C, chromosome-territory analysis, and single-cell Hi-C used to quantify architectural rescue. A concrete and realistic path to experimental verification of the prediction is proposed. Because lamina dysfunction is associated with aging and progeroid phenotypes, the result may contribute to the fundamental understanding of the aging process, including how LAD–lamina interactions contribute to aging-associated 3D genome disorganization.

We have also found that cell-to-cell conformational heterogeneity increases with lamin depletion and is largely restored toward WT values when LAD–lamina interactions are restored. This prediction, which can be tested using standard single-cell Hi-C experiments, contributes to our understanding of aging and may help explain, at least in part, why the epigenetic state of older differentiated cells is often less well defined than that of younger cells, a proposed hallmark of epigenetic aging[99]. Significant loss or dysfunction of lamins during aging or disease [11, 58, 69] can contribute to increased disorder in gene expression by enhancing variability in the global chromatin architecture. Notably, the rise of cell-to-cell conformational heterogeneity upon lamins depletion is largely reversible.

Thus, the youthful 3D genome architecture may be encoded not in a unique conformation, but in a distributed interaction landscape, including LAD–lamina contacts, TAD–TAD affinities, nuclear confinement, chromosome topology, etc. If aging degrades some of these organizing interactions without erasing the underlying genomic addresses, then restoring the interactions may allow chromatin to self-organize back into a youthful ensemble. Given the connection between lamin dysfunction and aging-associated phenotypes, we suggest that restoring functional LAD–lamina interactions may restore young-like 3D genome architecture and may be worth testing for effects on organismal aging phenotypes in fruit fly.

A methodological advance that greatly facilitated our work is the notion of a Shannon entropy for Hi-C maps that we have introduced and developed here. The new quantity is introduced as a measure of the organization/disorganization of chromatin conformational states as defined by its Hi-C map, and is designed to preserve the key features of entropy such as additivity for non-interacting systems. Throughout the work, we have used the Hi-C entropy as a simple and convenient metric of conformational disorder induced by age-associated perturbations such as lamins depletion. We have demonstrated that the new metric is sensitive to even subtle relevant changes in the Hi-C map, *e.g.*, it can robustly distinguish between experimental WT and *lamins depleted* Hi-C maps of *Drosophila melanogaster*, even though the visual differences are barely discernible. The limited testing is encouraging: Hi-C entropy is higher in the Progeria example analyzed here and tends to increase in selected chromosomes from human and mouse cerebellar cells across the lifespan. Importantly, the numerical differences in Hi-C entropy values upon lamins depletion predicted in our simulations of the chromatin evolution upon lamins depletion agree well with those estimated from the corresponding experimental Hi-C maps, supporting the model’s use in this context.

The work has several limitations. First, near-restoration of selected 3D chromatin-architecture metrics has so far been demonstrated only in simulations of a female fruit fly nucleus, which has a relatively simple composition of only 4 chromosome pairs. Although the fruit fly is a higher eukaryote whose genome organization is complex, it is less complex than that of mammals with considerably more chromosomes. When our key conclusions are tested in mammals, this additional complexity of the 3D genome organization may result in the type of conformational rejuvenation described here being less complete and/or slower. A related methodological limitation is that models of chromatin considered here are at the TAD resolution – the resulting computational efficiency of the simulation allowed us to simulate a biological ensemble of fruit fly nuclei on time scales of the interphase. Another limitation of the work is that we have only investigated interphase chromatin, that is, no passage through full cell cycle was considered. This limitation partly stems from the computational model used[102], which simulates only interphase chromatin, and partly from the limited availability of high-quality Hi-C maps covering entire lifespans. Our focus on the interphase is motivated in the Introduction, where we have argued that epigenetic rejuvenation approaches can benefit from avoiding DNA replication and the associated errors. Finally, we stress that despite encouraging results, we are not proposing Hi-C entropy as a *bona fide* epigenetic clock. Epigenetic clocks are complex, often relying on multiple features: further exploration is needed to determine whether the notion of Hi-C map entropy can be a useful addition to the existing arsenal of such features, or be useful as a stand-alone metric of chromatin conformational age.

## Figures and Tables

**Table 1:** Comparison of entropies of the four idealized Hi-C matrices described in Fig. 1. The last row is added for completeness.

System	*N*	*S* (bits)

(a) Uniform distribution	1169	11909
(b) Strong interactions	1169	10894
(c) Weak interactions	1169	9540
(d) Freely jointed chain	1169	7746
Fully diagonal contact matrix	1169	0

**Table 2:** Whole-genome mean conformational heterogeneity (averaged over *s*) for each state. Values are reported as unweighted mean ± jackknife standard error across replicate Hi-C maps (*n* = 18 per state) and are ordered from highest to lowest.

State	$\bar{\mathrm{CH}}$ (×10^−5^)

*heat death*	9.35 ± 0.46
*lamins depleted*	6.78 ± 0.24
Rejuvenated from *lamins depleted*	6.60 ± 0.32
WT	6.29 ± 0.24
Rejuvenated from *heat death*	6.15 ± 0.47

## Footnotes

a On the timescale of ~ 3 hrs, Fig. 4, we have not observed signs of metastability[14] of the recovered WT state, although we can not exclude the possibility that it might be present on timescales shorter than the temporal resolution (10 mins) of our computational experiment. A direct comparison with Ref. [14], which reported signatures of a metastable state (hysteresis), is difficult for several reasons, including difficulty in comparing the relevant timescales, and the fact that we have simulated the entire fruit fly nucleus, while a single human chromosome was modeled in Ref[14].

b This limiting *heat death* state is conceptually related to the “desorbed-extended” state described in simulations of a human chromosome interacting with a model nuclear lamina[14]. In that model, simultaneous weakening of heterochromatin–lamina and heterochromatin–heterochromatin interactions produced an extended chromosome organization associated with progeroid cells. It is worth noting that the major loss of structure seen in the Hi-C map upon the *WT* → *heat death* transition in our simulation, Fig. 6, is qualitatively similar to that in the *WT* → *Progeria* transition, see Fig. 2 and Ref.[14]. We refrain from further quantitative comparison here because the *heat death* state is more extreme (all attractive TAD–TAD, LAD–NE, and nucleolar interactions are set to zero), and the simulation is performed for a whole-nucleus *Drosophila* genome ensemble rather than for a single human chromosome.
