# Supplementary material for: Reversing aging-like 3D genome disorganization in a Drosophila interphase model
Source: bioRxiv. 2026 Oct 7:2026.07.16.739007. Originally published 2026 Jul 21. Preprint. [Version 2] doi: 10.64898/2026.07.16.739007 (PMC13419808; doi:10.64898/2026.07.16.739007)
Supplement: Supplementary file 2 [file media-2.zip › Supplemental Movies/README.pdf]

Supplemental Movies For: Reversing aging-like 3D genome disorganization in a *Drosophila* interphase model

Alexey V. Onufriev* [1,2,3], Junkai Zhang[2,5], Igor V. Sharakhov[3], and Igor S. Tolokh[4]

[1]Departments of Computer Science, Virginia Tech
[2]Department of Physics, Virginia Tech
[3]Center for Soft Matter and Biological Physics, Virginia Tech
[4]Department of Entomology, Virginia Tech
[5]University of Illinois, Chicago

July 2026

*Correspondence: alexey@cs.vt.edu

The dynamic model of the fruit fly nucleus.

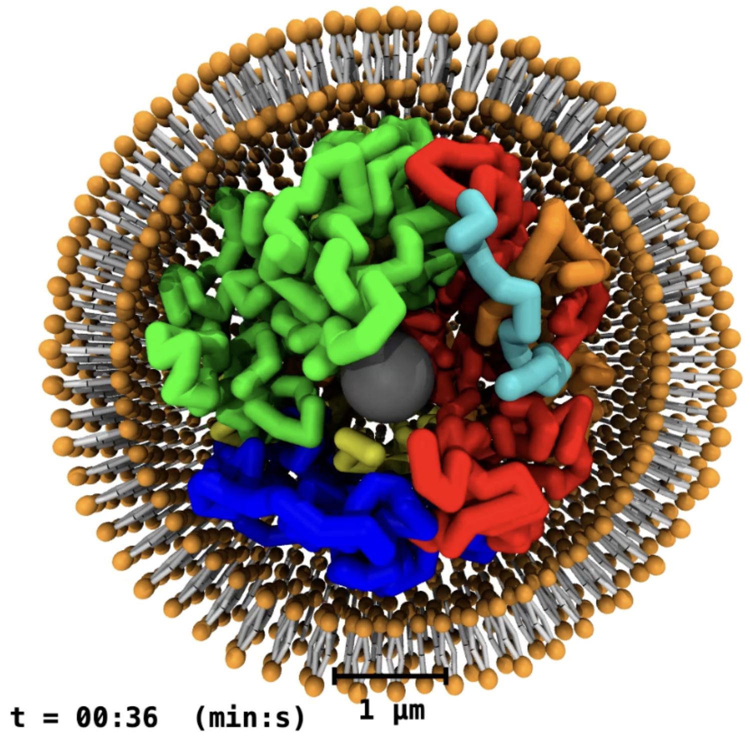

Figure SMovie1: A movie illustrating about 30 seconds of the computed temporal evolution of the entire fruit fly chromatin confined within the nucleus during interphase. Only one out of four experimentally observed mutual arrangements (CYSX-6S[1]) of the chromosomes is shown. Each chromosome arm is colored differently, the gray sphere is the nucleolus.

Recovery from *lamina-depleted* state

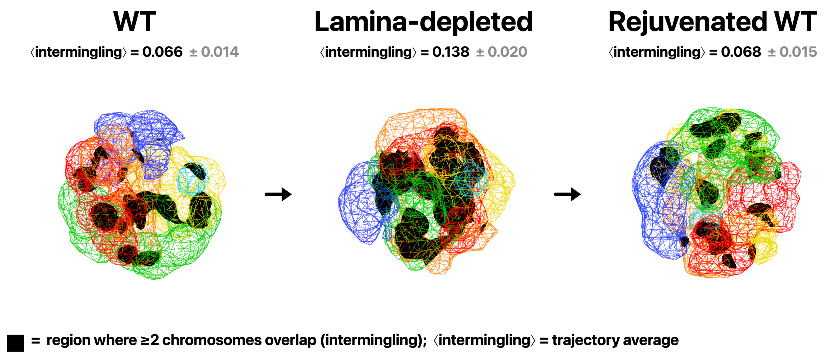

Figure SMovie2: Temporal evolution of chromosome territories along the WT → *lamina-depleted* → WT rejuvenation pathway (about 10 seconds per state). The intermingling between chromosomes increases following transition to the *lamina-depleted* state, and returns to its WT value upon the restoration of the attractive interactions between LADs and the nuclear envelope (NE), present in the WT state and absent from the *lamina-depleted* state. The intermingling regions are colored black. Each chromosome arm is colored differently, the gray sphere is the nucleolus. For clarity, only one out of four experimentally observed mutual arrangements (CYSX-6S[1]) of the chromosomes is shown.

Recovery from " *lamina-depleted + compartment-disrupted* " state

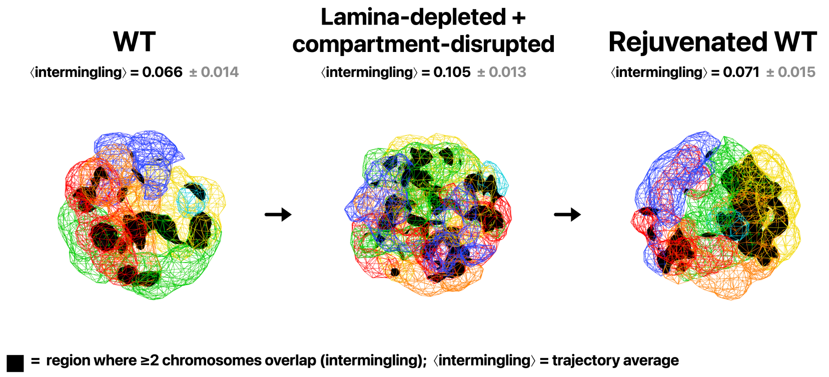

Figure SMovie3: Temporal evolution of chromosome territories along the WT → *lamina-depleted + compartment-disrupted* → WT rejuvenation pathway (about 10 seconds per state). The intermingling between chromosomes increases following transition to the *lamina-depleted + compartment-disrupted* state, and returns to its WT value upon the restoration of the attractive interactions between the TADs, and between LADs and the nuclear envelope (NE), present in the WT state and absent from the *lamina-depleted + compartment-disrupted* state. The intermingling regions are colored black. Each chromosome arm is colored differently, the gray sphere is the nucleolus. For clarity, only one out of four experimentally observed mutual arrangements (CYSX-6S[1]) of the chromosomes is shown.

References

[1] Igor S Tolokh, Nicholas Allen Kinney, Igor V Sharakhov, and Alexey V Onufriev. Strong interactions between highly dynamic lamina-associated domains and the nuclear envelope stabilize the 3d architecture of drosophila interphase chromatin. *Epigenetics & Chromatin*, 16(1):21, 2023.
